# *In Vitro* Maturation of Oocytes in Women at Risk of Ovarian Hyperstimulation Syndrome-A Prospective Multicenter Cohort Study

**DOI:** 10.22074/ijfs.2019.5452

**Published:** 2019-01-06

**Authors:** Sanne C. Braam, Dimitri Consten, Jesper M.J. Smeenk, Ben J. Cohlen, Max H.J.M. Curfs, Carl J.C.M. Hamilton, Sjoerd Repping, Ben W.J. Mol, Jan Peter de Bruin

**Affiliations:** 1. Department of Obstetrics and Gynaecology, Academic Medical Center, Amsterdam, The Netherlands; 2. Department of Obstetrics and Gynaecology, St. Elisabeth Hospital, Tilburg, The Netherlands; 3. Department of Obstetrics and Gynaecology, Isala Clinics, Zwolle, The Netherlands; 4. Department of Obstetrics and Gynaecology, Jeroen Bosch Hospital, ‘s-Hertogenbosch, The Netherlands; 5. Department of Obstetrics and Gynaecology, Monash University, Clayton, Victoria, Australia

**Keywords:** *In Vitro* Maturation, Polycystic Ovarian Syndrome, Prospective Study

## Abstract

**Background:**

*In vitro* maturation (IVM) is an artificial reproductive technology in which immature oocytes are harvested from the ovaries and subsequently will be matured in vitro. IVM does not require ovarian hyperstimulation (OH) and thus the risk of ovarian hyperstimulation syndrome (OHSS) is avoided. In this study, we assessed the live birth rate per initiated IVM cycle in women eligible for in vitro fertilization/intracytoplasmic sperm injection (IVF/ ICSI) and at risk for OHSS. Furthermore, we followed women who were not pregnant after IVM and committed to a conventional IVF/ICSI procedure.

**Materials and Methods:**

In this multicenter prospective cohort study, we started 76 IVM cycles using recombinant follicle stimulating hormone (rFSH) priming in 68 patients. There were 66 oocyte retrievals, in which a total of 628 oocytes were collected. We incubated the immature oocytes for 24-48 hours and fertilized those that reached metaphase II by ICSI.

**Results:**

Three hundred eighty six (61% oocytes) achieved metaphase II. The fertilization rate was 55%. We performed 59 embryo transfers (1.9 embryos per transfer) in 56 women, including 3 frozen embryo transfers. There were four ongoing pregnancies (5.3% per initiated cycle) leading to the birth of a healthy child at term. None of the patients developed OHSS. The ongoing pregnancy rate of the first conventional IVF/ICSI cycle after an unsuccessful IVM cycle was 44%, which was unexpectedly high.

**Conclusion:**

We concluded that IVM led to live births but with low effectiveness in our study. Earlier reported IVM success rates are higher which can be caused by a more extended experience in these centers with the intricate laboratory process. However, a possible selection bias in these studies cannot be ruled out. Furthermore, IVM might have a beneficial effect on further IVF/ICSI treatments due to its “ovarian drilling” effect.

## Introduction

In vitro maturation (IVM) is an artificial reproductive technology which involves the retrieval of immature oocytes from the ovaries. Subsequently, these oocytes are matured in vitro. Since IVM does not require ovarian hyperstimulation (OH), the risk of ovarian hyperstimulation syndrome (OHSS) is avoided. Other potential benefits of IVM may include patient friendliness and reduced costs when compared to a conventional in vitro fertilization (IVF) treatment.

The first report of a pregnancy and childbirth after IVM was published in 1991 (1). It was estimated in 2012 that over 3,000 children were born after IVM worldwide. Clinical pregnancy rates per embryo transfer considerably vary from 4-53% (2-4). Also, different IVM techniques are applied with regard to the administration of human chorionic gonadotropin (hCG) and recombinant follicle stimulating hormone (rFSH) priming. Furthermore, indications for IVM treatment have expanded in recent years including oocyte donation and fertility preservation (5-9).

It is hard to value the success rates of the published cohort studies so far. In most studies, the criteria for patient selection are not clarified and no information is given on previous infertility treatments in selected patients (10, 11). Furthermore, studies often report pregnancy rates per oocyte pickup or even per embryo transfer and not per started cycle. It is therefore hard to counsel future patients. Will they benefit from IVM? Or will they stand a better chance with another medically assisted reproductive technique?

In this study, we aimed to establish the live birth rate with IVM in a well-defined and prospectively registered population of women with an indication for IVF or intracytoplasmic sperm injection (ICSI) that were at risk of OHSS. Furthermore, the follow up of the children born after IVM is described and the follow up of patients with regard to their subsequent fertility treatments.

The goal of this study was to introduce IVM as a novel technique in the Netherlands and to continue with a randomized trial comparing pregnancy rates for IVM and IVF.

## Materials and Methods

This multicenter prospective cohort study was performed in three non-academic hospitals in the Netherlands: Jeroen Bosch Hospital, St Elisabeth Hospital, and Isala Clinics. All participants provided written informed consent. The study was approved by the Central Committee on Research involving Human Subjects (CCMO NL29051.000.09) and by the board of each participating hospital. All initiated IVM cycles were registered prospectively.

### Subjects

Patients were eligible when at least one previous IVF cycle had been complicated by OHSS or cancelled because of imminent OHSS. Also, PCOS patients with an indication for IVF because of failure to achieve an ongoing pregnancy after regular treatments of ovulation induction (OI) with clomiphene citrate, laparoscopic ovarian drilling and rFSH with or without intrauterine inseminations (IUI) could enter the study. PCOS was diagnosed according to the Rotterdam criteria (12, 13). Patients had to be between 18 and 38 years of age.

### Introduction of *in vitro* maturation technique

To prepare the staff for implementing the IVM technique two clinicians and two embryologists visited the Väestöliitto (The Family Federation of Finland) Fertility Clinic, Helsinki, Finland and the Biogenesi Reproductive Medicine Centre, Istituti Clinici Zucchi, Monza, Italy. Protocols were studied and discussed, and all different treatment steps were practiced. Next, mock cycles with immature oocytes of regular IVF and ICSI cycles were practiced in our Dutch laboratories. Then, the proof of principle cycles was started with consenting couples.

The prospective cycles reported in this study were not started before we proved to be able to achieve ongoing pregnancies.

### Cycle monitoring and oocyte retrieval

Cycle monitoring and oocyte retrieval were based on the ‘Monza-protocol’ (7). At the start of the treatment, a baseline ultrasound was performed on cycle day 2, 3 or 4. In patients with severe oligomenorrhoea (cycle length >42 days) or amenorrhoea, a withdrawal bleeding was induced with 7 days 10 mg progestagen (Provera®, Pfizer) orally. The cycle was excluded if at baseline ultrasound the endometrial thickness exceeded 4 mm or an ovarian cyst larger than 12 mm was present.

Subsequently, ovarian priming was performed by the administration of 150 IU rFSH s.c. (Puregon®, Merck Sharp & Dohme) on cycle day 3 to 5. The second ultrasound was scheduled at cycle day 6 to 8 and thereafter at one- or two-day intervals until the identification of a dominant follicle. A dominant follicle was defined as a follicle that had grown to at least 8 mm (but not larger than 12 mm) accompanied by a thickening of the endometrial lining to 5 mm or more (14). Subsequently, 10.000 IU of hCG (Pregnyl®, Merck Sharp & Dohme) were administered subcutaneously and the oocyte retrieval was scheduled 38 hours later (15). A cycle was cancelled when there was a follicle larger than 14 mm or an endometrium less than 5 mm. Oocytes were retrieved by transvaginal ultrasound-guided needle aspiration at a vacuum pressure of 80-100 mmHg with a 16 gauge needle (Origio).

### *In vitro* maturation and embryo culture

In the fertility laboratory, the oocytes were isolated from the punctate using a cell strainer filter and transferred to IVM culture medium (Medicult IVM®, Origio). This medium was supplemented with 100 mIU/ml hCG, 75 mIU/ ml FSH, and 10% protein solution (GPO, Sanquin, the Netherlands).

Subsequently, the collected oocytes were incubated (at 36.8 Celsius and 5.2% CO_2_) in this medium for 24-48 hours to induce final oocyte maturation. Oocytes reaching the metaphase II stage were fertilized using ICSI. In cases in which a metaphase II oocyte already was present at oocyte retrieval, this oocyte was injected with the husband’s sperm the same day (16). After ICSI, the oocytes were transferred to Human Tubal Fluid (HTF, Lonza, Belgium) with 8,8% protein solution (GPO, Sanquin, the Netherlands) and incubated at 36.8-37.0°C and 5.0-5.2% CO_2_. Embryo morphology was assessed daily from day 1 up to day 5, based on the cell number and overall appearance of the embryo (good, average, poor) considering fragmentation, equality of the blastomeres and multinucleation. A good embryo has 0-20% fragmentation, equal blastomeres, and no multinucleation. An average embryo has more than 20% fragmentation but not 50%, or unequal blastomeres, but no multinucleation. A poor embryo has more than 20% fragmentation and unequal blastomeres or more than 50% fragmentation or is multinucleated. For embryo transfer, the best available embryo or embryos were selected based on the above-mentioned criteria.

### Luteal support and embryo transfer

At the day of oocyte retrieval, luteal support was started with Estradiol (Progynova®, Bayer) 2 mg orally three times a day and Progesterone (Utrogestan®, Besins International) daily vaginally 600 mg started on the day after the oocyte retrieval. In cases with a positive pregnancy test, oestrogen and progesterone supplementation were continued until ten weeks of gestation.

Embryo transfer was conducted on day three or day four after ICSI and a maximum of two embryos were transferred per cycle. Whenever an embryo was available, it was transferred, without again determining endometrial thickness or endocrine parameters. Remaining embryos were selected for cryopreservation according to the standard IVF/ICSI procedures and criteria.

### Outcome

The primary endpoint of the study was the live birth rate per started cycle. Secondary endpoints of the study were antral follicle count at the start of the IVM treatment cycle, number of retrieved oocytes per cycle, number of metaphase II oocytes at retrieval, maturation rate of oocytes, fertilisation rate of mature oocytes, number and quality of embryos, clinical pregnancy rate, live birth rate per oocyte retrieval and per embryo transfer, and the number and nature of adverse events during or following IVM/ICSI. Further endpoints were the health and development of IVM/ICSI children during a two-year follow- up program and the ongoing pregnancy rate of women who continued with IVF after unsuccessful IVM.

Live birth rate was defined as the birth of a living child beyond 24 weeks of gestation. Clinical pregnancy was defined by the ultra-sonographic presence of a gestational sac, four weeks after embryo transfer. Ongoing pregnancy was defined by the ultra-sonographic presence of a vital embryo eight to ten weeks after embryo transfer.

### Paediatric follow up

To monitor the safety of the IVM technique, children were evaluated after birth and at ages of 6 months, 1 and 2 years. Follow up consisted of an evaluation on the following domains using internationally accredited and validated tests: motor development, cognitive development, and behaviour [Alberta Infant Motor Scale (AIMS), Bayley scale of infant development III (BSID III), Movement ABC-II, Wechsler Preschool and Primary Scales of Intelligence].

### Statistical analysis

We calculated the percentage of ovum pick-ups, embryo transfers, pregnancies, and live birth per cycle; both for the IVM group, as well as for the subsequent IVF group. Continuous variables were presented as the mean, median (including range) or percentage where appropriate. Differences in the variables of IVF treatments prior to and after IVM were analysed with the Wilcoxon Signed Rank Test. Analyses were performed using the Statistical Package for the Social Sciences 22.0 software for Windows.

### Sample size

This cohort study was designed as a pilot for starting a randomized controlled trial of IVM versus conventional controlled ovarian hyperstimulation (COH)/IVF or COH/ ICSI. Therefore, we had to establish the live birth rate of IVM in our selected population. We expected the live birth rate of conventional COH/IVF or COH/ICSI in this group to be 15% per started cycle. We argued that IVM, to be a reasonable alternative to the conventional techniques and worthwhile to stand in a direct comparison in a trial comparing 2 IVM cycles to one conventional IVF or ICSI cycle, should at least have a mean live birth rate of 7.5% in 75 started cycles.

## Results

### Participants

Between May 2010 and October 2011, we included 68 subfertile women. In these women, we conducted 76 IVM cycles. The mean female age was 29.8 ± 3.9 years (mean ± SD) and the mean duration of subfertility was 2.7 ± 1.6 years. The main primary diagnoses were PCOS (n=32) and male subfertility (n=29, Table 1). 

**Table 1:** Baseline characteristics (n=68)


Variable		n

Age (Y)		29.8 ± 3.9
BMI (kg/m^2^)		24.6 ± 4.74
Subfertility couple		
	Primary	44 (65)
	Secondary	24 (35)
Duration subfertility (Y)		2.7 ± 1.6
Primary diagnosis		
	Cycle disorder	32 (47)
	Male subfertility	29 (43)
	Other	7 (10)
Previous fertility treatment		
	None	5
	Ovulation induction with anti-estrogens and gonadotropins	18
IVF or ICSI cycles		
	1	22
	2	15
	3 or more	8


Data are presented as mean ± SD or n (%). BMI; Body-mass index, IVF; In vitro fertilization, and ICSI; Intracytoplasmic sperm injection.

### Previous fertility treatments

Most women had previously received assisted reproductive therapy (n=63, 91%). There were 18 women who had undergone OI with or without IUI, while 45 women had at least one previous IVF or ICSI cycle (Table 2). They started a total of 81 IVF cycles of which 41 were cancelled. Of these 41 cancelled cycles, 32 cycles were cancelled because of imminent OHSS and 9 cycles were cancelled because of insufficient follicle growth, mainly following a previous cancelled cycle of imminent OHSS. The remaining 40 cycles were completed but did not result in pregnancy.

**Table 2:** Cycle and laboratory data of 76 in vitro maturation cycles in 68 women


Variable	n or n (%)

Started cycles	76
Oocytes collections	66
Antral follicle count (mean)	30 (range 9-80)
Dominant follicle size (mean)	11 (range 8-14) mm
Endometrial thickness at the time of oocyte collection (mean)	8 (range 4-15) mm
Oocytes retrieved	628
Retrieved oocytes per oocyte collection (mean)	9.5 (range 0-29)
Mature oocytes at the time of oocyte collection (%)	34 (5)
Mature oocytes available and inseminated (%)	386 (61)
Fertilized eggs (%)	212 (55)
Embryos	197
Embryos per retrieval (mean)	3


**Fig.1:**
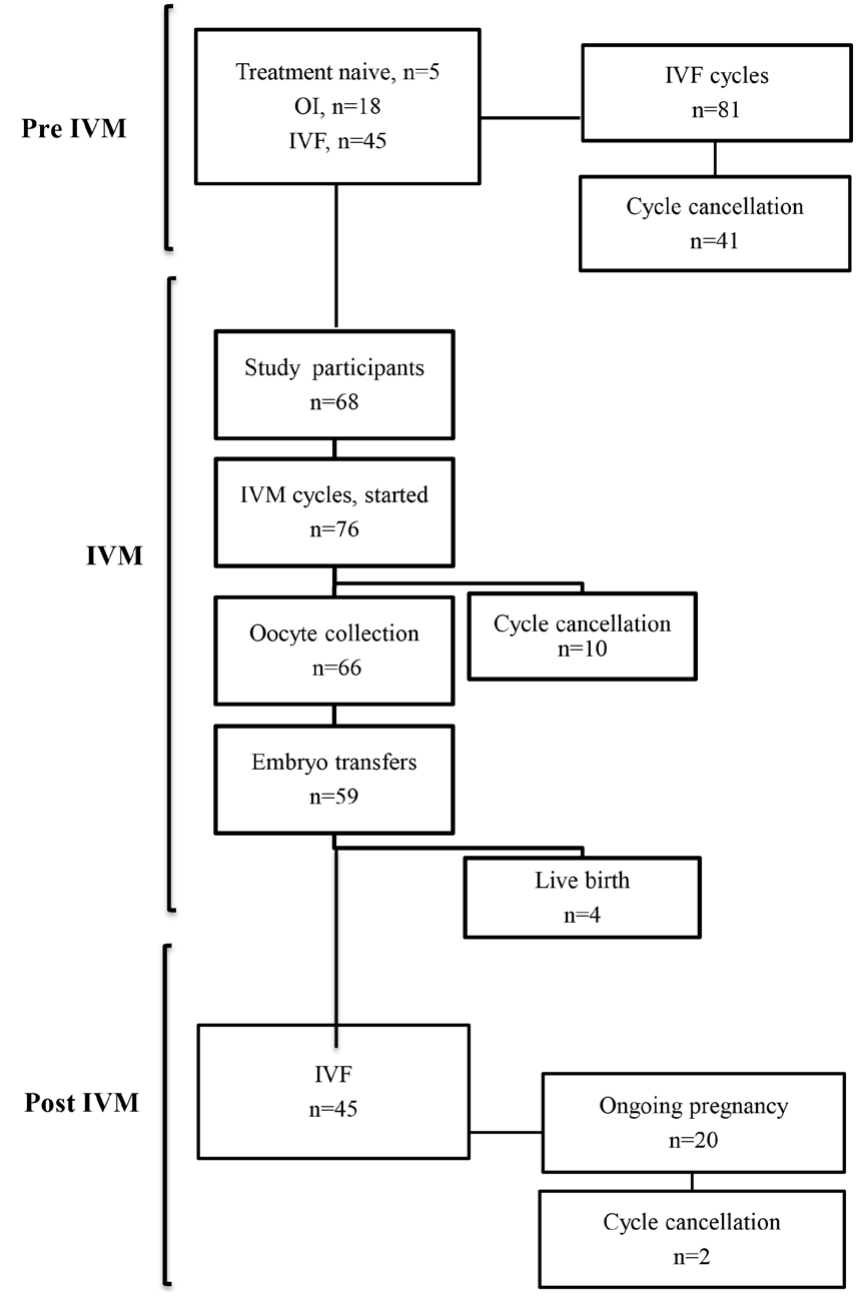
Representative scheme showing the patient flow, including previous and subsequent treatment cycles. IVM; In vitro maturation, IVF; In vitro fertilization, and OI; Ovulation induction.

Five patients were treatment-naive when entering the study. These were patients with a combined diagnosis of PCOS and severe male subfertility leading to an indication for ICSI (Fig .1).

### *In vitro* fertilization treatments

The mean antral follicle count at the start of the IVM cycle was 30. Of 76 started IVM cycles, 10 cycles were cancelled because of inadequate endometrial response, meaning an endometrial thickness of less than 5 mm at the time that follicular dominance was seen at the transvaginal ultrasound. In the remaining 66 cycles, a total of 628 oocytes were collected (range 0-29 oocytes per oocyte retrieval). Of these, 5% already were at metaphase II at oocyte retrieval, 56% reached metaphase II after 24-28 hours of maturation. In total 61% (386 oocytes) achieved metaphase II (range 0-17 oocytes per oocyte retrieval). The fertilization rate was 55% (212 embryos). 59 embryo transfers (mean of 1.9 embryos per transfer) were performed, including 3 frozen embryo transfers (Table 2). The quality of the transferred embryos is described in Table 3.

Six pregnancies occurred, of which four were ongoing (5.3% per initiated cycle). One of the ongoing pregnancies developed from a frozen embryo transfer. All ongoing pregnancies led to the birth of a healthy child at term. None of the patients developed OHSS. The live birth rate per OPU was 6%, the live birth rate per embryo transfer was 10%.

**Table 3:** Embryo transfer, embryo quality, and clinical outcome


Variable		n

Embryo transfers		59 (3 frozen embryo transfers)
Transferred embryo’s (mean)		1.9
Embryo quality at transfer (%)		

Good	51
Average	40
Poor	9
Positive β-hCG tests		6
Clinical pregnancies		5
Live births		4
Live birth rate per started cycle (%)		5.3
Multiple pregnancies		0


ß-hCG; Human chorionic gonadotropin.

### Subsequent in vitro fertilization cycle

Of 64 patients with an unsuccessful IVM treatment, 45 proceeded to an IVF cycle. The treatment protocol consisted of a gonadotropin-releasing hormone (GnRH) agonist or antagonist scheme, and a daily dose of rFSH of 75 to 225 IU, followed by hCG triggering (10.000 IU) and luteal support with progesterone. The number of cancelled cycles was two (4%). OHSS was the reason for cancelling one cycle. In total, 44% (20/45) of these patients achieved an ongoing singleton pregnancy in their first IVF treatment after IVM. Of them, 36% (16/45) was pregnant after a fresh embryo transfer and four pregnancies resulted from cryo-transfers of embryos from the first IVF cycle after IVM (Fig .1).

**Table 4:** Cycle and laboratory characteristics of conventional IVF/ICSI cycles before and after the studied IVM cycles


Variable		IVF before IVM n=23	IVF after IVM n=20	P value

Stimulation protocol (n)				
	Agonist	19	14	
	Antagonist	3	2	
	Other^a^		4	
Insemination (n)				
	Conventional	10	9	
	ICSI	13	11	
Starting dose gonadotrophin (IU, median, range)		150 (75-225)	125 (75-225)	NS
Total gonadotrophin (IU, median, range)		1316.5 (750-4900)	1200 (600-2250)	NS
Number of dominant follicles (≥12 mm, median, range)		18 (1-42)	12 (2-25)	0.003
Number of collected oocytes (median, range)		9 (3-24)	7 (3-21)	NS
Number of embryos (median, range)		6 (2-16)	3 (1-16)	NS
Number of transferred embryos (median, range)		1.0 (1-2)	1.0 (1-2)	NS


IVF; *In vitro* fertilization, ICSI; Intracytoplasmic sperm injection, IVM; In vitro maturation, NS; Not significant, and ᵃ; Clomiphene citrate.

Of these patients, we aimed to compare the cycle characteristics of the IVF cycle before and after the IVM treatment (Table 4). Details of gonadotropin dosage and the number of follicles were available in 23 patients. Gonadotropin dosage was determined according to the local protocol which allowed dose adjustments considering an observed response to earlier ovarian stimulation. Overall, there was a small but insignificant difference in the starting dose and the total dose of gonadotropins. The number of dominant follicles was lower in IVF cycles performed after IVM (18 vs. 12, P=0.003) but that did not result in a lower number of collected oocytes. 

Furthermore, there were three cases of spontaneous conceptions among the remaining women with polycystic ovary syndrome (PCOS)-related anovulation. In these patients, spontaneous ovulation and conception occurred within four months after IVM treatment.

### Follow up of children

All four IVM children were delivered at term (Table 5). One was large for gestational age, and the other three had an appropriate birth weight. No congenital malformations were present. The follow up showed normal physical growth for all children. Two children were in the normal to high range on the various motor, cognition, and behaviour scales. Motor development was slow in two children (AIMS <5^th^ percentile and BSID-II development index of <55, respectively).

**Table 5:** Obstetric outcomes of children born after IVM treatment


Gestational age (weeks)	Mode of delivery	Sex	Birth weight (g and P value*)

42+3	Caesarean section	Male	4870 (>p97.7)
41+3	Vacuum extraction	Male	4105 (p80-p84)
39	Spontaneous	Female	3290 (p20-p50)
41	Caesarean section	Male	4200 (p84-p90)


*; The Netherlands Perinatal Registry Birth weight centiles and SD, www.perinatreg.nl., and IVM; In vitro maturation.

## Discussion

We were able to introduce IVM in the Netherlands, accomplishing successful pregnancies and the birth of healthy newborns. We selected a group of patients with increased risk of OHSS and in none of the IVM cycles OHSS occurred. The live birth rate per started cycle was limited to 5.3%. This percentage is low compared to the results previously published. In other studies, however, pregnancy rates per started cycle are not always available, mostly pregnancy rates per oocyte retrieval or embryo transfer are reported. Also, the maximum number of embryos per transfer in our study was limited to two, while other studies report transfers of up to four embryos per cycle (16). Furthermore, as already mentioned in the introduction section, in most previous studies patient characteristics and the previous fertility treatments are not revealed. Therefore, it is difficult to compare the results in our group of patients with the results of others. Possibly, the a priori chance of pregnancy was reduced in our patients as the majority already underwent several unsuccessful treatment cycles of OI, IUI and/ or IVF.

Apart from differences in patient characteristics, there is a lot of variation in how IVM is performed. IVM can vary from not using any gonadotropins at all, using FSH priming, triggering with hCG, or using both FSH and hCG. Nowadays, most research groups use FSH priming as it improves the yield of competent oocytes. The use of hCG triggering, however, is more controversial, as it may lead to maturation of oocytes in vivo. This can result in a mix of immature and also mature oocytes at oocyte retrieval. Some argue, therefore, that using hCG is not compatible with the true definition of IVM. Accordingly, cycles with hCG-use should be distinguished and renamed as “natural cycle IVF” or “truncated IVF” (17, 18). 

Indeed, in our study, with FSH priming and the administration of hCG 38 hours prior to ovum pick up a small proportion of 5% metaphase II oocytes were retrieved. It is not likely that this had a large influence on our success rate. A recent systematic review could not find a significant difference in live birth rates in hCG versus non-hCG IVM cycles (19). 

In two recent cohort studies, a comparison between IVM and IVF in PCOS patients was made. Walls et al. (4) showed a lower clinical pregnancy rate in the IVM group. There were significantly fewer live births resulting from IVM treatment for both fresh and cumulative cycle outcomes. However, there was no difference in live birth rates resulting from frozen embryo transfers between IVM and IVF treatment. Das et al. (20) compared IVM with a more novel GnRH agonist trigger IVF protocol. The latter protocol has been shown to result in lower OHSS rates than IVF protocols with hCG triggering (21). Both IVM and IVF with GnRH-antagonist protocol seem to be effective treatment regimens in women with PCOS. Although IVM was associated with a lower risk of OHSS, the live birth rate was significantly higher in IVF with GnRH agonist triggering. Also, Gremeau and colleagues reported the higher implantation and pregnancy rates in conventional IVF using a long GnRH agonist protocol (22).

At the time of our study, the GnRH agonist protocol was the prevailing method in the Netherlands. In the present study, the follow-up data of subsequent IVF treatments of 44% ongoing pregnancies per started cycle were highly favourable. This is remarkable considering the poor results of the IVF cycles in these patients prior to the IVM treatment cycles, which were characterized by very high cancellation rates. On the one hand, this can be explained by FSH dose adjustments. However, in our study, the mean starting dose and total dose of FSH of the cycle following IVM treatment was not significantly different from the dose in the IVF cycle preceding IVM. On the other hand, the oocyte retrieval in IVM may induce a change in the ovaries comparable to the result of laparoscopic ovarian drilling. This is compatible with our finding of significantly fewer follicles = 12 mm in the IVF cycle following IVM. Furthermore, we have described spontaneous cycle restoration and spontaneous pregnancies in a case series of three patients following IVM treatments (23). Also others have reported improved outcomes in IVF cycles that were preceded by IVM (24, 25). Findings of transient but significant changes in ovarian endocrine parameters after the retrieval of immature oocytes in patients with PCOS could be a possible biological plausibility for this (26, 27).

The limitations of our study were in the cohort study design and the number of studied patients. However, the sample size of the study was comparable with other previous studies (2, 11). Also, an overlap of patients in some of the previous reports cannot be excluded.

As IVM is considered a novel technique in the Netherlands, a learning curve has been probably interplayed with the final study results, although the maturation rates and fertilization rates were comparable with other IVM studies. Thus far, only a small number of research groups seem to achieve high pregnancy rates (3, 28). These research groups may be able to harvest and process oocytes faster, which sustains viability. Also, subtle adjustments in culture protocols optimizing culture media, incubator temperature or CO_2_ concentration can play a role.

Strengths of our study were the inclusion of a well-defined patient group. Although we included patients with different fertility diagnoses, they all were characterized by an increased risk of OHSS and eligible for IVF or ICSI. All patients and all IVM cycles including cancelled cycles were recorded prospectively in a central study register. Also, this is the first study in which all data are reported on fertility treatments prior to and after the IVM study cycle, as well as the follow up of the IVM-children up to two years of age.

In this study, no malformations were found. Although all children were thriving, in two of four children, motor development was slow on validated tests. In several reviews, the authors have proposed that IVM is not associated with an increase in numbers of congenital malformations (29, 30). However, more subtle developmental differences cannot be ruled out. A French cohort of IVM children, for example, showed a higher mean weight in girls at one year of age (31). We should consider that data on IVM children are limited, both in the number and duration of the follow-up (32). Further monitoring of these infants outcomes is required.

## Conclusion

We concluded that IVM led to live births but with low effectiveness in our study. Based on the data presented in this article, the national medical ethical committee did not allow IVM to be continued in the Netherlands and we had to cancel our plans to conduct an a randomized controlled trial (RCT) of IVM versus IVF. According to a Cochrane review, there is still no evidence from randomized controlled trials upon which to base any practice recommendations regarding IVM before IVF or ICSI for women with PCOS. Thus randomized control trials comparing IVM with IVF are needed for a more exact estimate of the effectiveness of IVM in specific groups of patients. Finally, we would like to encourage other IVM researchers to reveal data on patient selection criteria in future publications.
